# Cortical spreading depression impairs hippocampal long-term potentiation by the alteration of glutamate receptor responses

**DOI:** 10.1186/1129-2377-14-S1-P66

**Published:** 2013-02-21

**Authors:** S Bongsebandhu-Phubhakdi, M Maneepark, A Srikiatkhachorn

**Affiliations:** 1 Department of Physiology, Faculty of Medicine, Chulalongkorn University, Thailand

## 

There is a relationship between migraine aura and amnesic attack. Cortical spreading depression (CSD), a phenomenon underlying migraine attack, may be responsible for hippocampus-related symptoms. However, the precise role of CSD on hippocampal activity has not been investigated. This study aimed to investigate the alteration of hippocampal long-term plasticity and basal synaptic transmission induced by repetitive CSDs. Male Wistar rats were divided into CSD and control groups. Repetitive CSDs were induced in vivo by topical application of solid KCl. Forty-five minutes later, the ipsilateral hippocampus was removed, and hippocampal slices were prepared for a series of electrophysiological studies. After CSD induction, SDs also appeared in the hippocampus. Repetitive CSDs led to a decrease in the magnitude of long-term potentiation (LTP) in the hippocampus. CSD also reduced hippocampal synaptic efficacy, as shown by a reduction of postsynaptic á-amino-3-hydroxy-5-methyl-4-isoxazolepropionic acid (AMPA) receptor responses. In contrast, postsynaptic N-methyl-D-aspartate (NMDA) receptor responses remained unchanged. In addition, there were no changes in paired-pulse profiles between the groups, indicating that CSD did not induce any presynaptic alterations. Despite of unaltered NMDA receptor responses, CSD elevated the ratio of GluN2A to GluN2B subunit of NMDA receptor (Glu2A/2B ratio). These finding suggest that a reduction of postsynaptic AMPA receptor responses and an increase of Glu2A/2B ratio are the mechanism responsible for the impaired hippocampal LTP that was induced by CSD.
